# Can circadian rhythm predict changes in neurocognitive functioning in bipolar disorder: protocol of a 12-month longitudinal cohort study based on research domain criteria

**DOI:** 10.1080/07853890.2023.2240422

**Published:** 2023-07-28

**Authors:** Huirong Luo, Xueqian Wang, Yinlin Zhang, Junyao Li, Renqin Hu, Zheng Zhang, Qian Liao, Xiaoxin Zhou, Wei Deng, Jian Yang, Qinghua Luo

**Affiliations:** a Department of Psychiatry, The First Affiliated Hospital of Chongqing Medical University, Chongqing, China; b Department of Laboratory Diagnostic, Chongqing KingMed Institute for Clinical Laboratory Co.LTD, Chongqing, China; c Department of Clinical Nutrition, The Third Affiliated Hospital of Chongqing Medical University, Chongqing, China; d Affiliated Mental Health Center & Hangzhou Seventh People’s Hospital, Zhejiang University School of Medicine, Zhejiang, China; e Research Center for Metabolic and Cardiovascular Diseases, The Third Affiliated Hospital of Chongqing Medical University, Chongqing, China

**Keywords:** Bipolar disorder, circadian rhythms, actigraphy, neurocognitive functioning, research domain criteria (RDoC)

## Abstract

**Introduction:** Bipolar disorder (BD) is a prevalent and disabling mental disorder characterized by disrupted circadian rhythms and impaired neurocognitive features, both of which fall under the major domains of Research Domain Criteria (RDoC). However, there is limited evidence regarding the interaction between circadian rhythms and long-term neurocognitive functioning. Therefore, this longitudinal cohort study protocol aims to explore whether circadian rhythm can predict changes in neurocognitive functioning over time in patients with BD.

**Methods:** This study adopts a longitudinal cohort design, aiming to recruit 100 BD patients in either depressive or remitted states. Participants will undergo evaluations from clinical, circadian rhythm, and neurocognitive perspectives at baseline, 6-month, and 12-month follow-ups, involving questionnaires, actigraphy, and computed neurocognitive tests. We will examine both cross-sectional and longitudinal associations between participants’ circadian rhythm patterns and neurocognitive functioning. Statistical analyses will employ Spearman correlation and mixed regression models.

**Discussion:** We anticipate that circadian rhythms may serve as predictors of neurocognitive functioning changes. The findings of this study could offer supplementary insights into BD pathophysiology, potential treatment targets, and prediction.

**Trial Registration:** This study has been registered with the Chinese Clinical Trial Registry under the registration code ChiCTR2200064922 on 21st October 2022.

## Introduction

Bipolar disorder (BD) is characterized by alternating manic/hypomanic episodes and depressive episodes, with an incidence of about 1% − 4% and suicide mortality 20-30 times higher than that in the general population [1,2]. As a significant biological feature, disrupted circadian rhythms have been highlighted by the International Society for Bipolar Disorders (ISBD). Among BD patients, delayed and disrupted chronotypes, changes in circadian rhythm gene expression, and abnormalities in cortisol and melatonin levels are commonly observed, showing correlations with prognosis, social functioning, and lithium response [3–6]. Considering that many studies have utilized subjective measurements, such as rhythm questionnaires and sleep diaries, objective measurements appear more reliable, including actigraphy, polysomnography, and the concentration of correlated hormones, especially melatonin and cortisol in serum, saliva, hair, and urine [7]. However, despite actigraphy being highly recommended for circadian rhythm evaluation, its limited application in long-term BD studies due to a scarcity of prospective designs has been noted [8].

Moreover, neurocognitive impairment, a distinctive feature of BD, has garnered significant attention due to its impact on the quality of life and long-term rehabilitation, contributing to BD being ranked as the second greatest cause of days out of role [1,9]. Commonly affected cognitive domains in BD encompass working memory, sustained attention, and executive functions [10]. While Lancet reviews have almost universally suggested that BD patients exhibit neuroprogressive characteristics resembling schizophrenia, previous longitudinal studies have yielded uncertain conclusions, highlighting the heterogeneity of cognitive phenotypes [1,2]. As BD staging and phenotyping of rhythmic and cognitive states gain popularity, the Research Domain Criteria (RDoC) has separately incorporated constructs related to circadian rhythms, attention, and working memory into arousal and regulatory systems, as well as cognitive systems, encouraging investigations into neurocircuitry and advancing future disorder classifications [11,12].

Interestingly, exploring circadian rhythm influence on neurocognitive functioning seems feasible with potential pathophysiological inspirations [13]. Recent studies focusing on Alzheimer’s and other neurodegenerative diseases have speculated on the involvement of seasonal cognitive plasticity and circadian rhythm disruption in neurocognitive functioning changes [14–17]. Additionally, a few pieces of evidence also support such an association with mental disorders [18]. For instance, rats subjected to chronic jet lag simulation demonstrated enhanced depressive behaviors and cognitive deficits [19]. Large-scale UK Biobank data revealed an association between disrupted circadian rhythmicity in mood disorders and cognitive function [20]. Moreover, the ‘desynchronization effect’ resulting from evening chronotypes and circadian disruption might render individuals vulnerable to cognitive and psychiatric challenges [21]. Furthermore, certain molecules are believed to play a role in both circadian rhythm disruption and neurocognitive impairment, mediated through mechanisms like immune-inflammatory activities [22,23].

Regrettably, up to this point, no study has been found that focuses on the long-term clinical correlation between circadian rhythm and neurocognitive functioning in BD patients. Exploring such a correlation holds utmost importance because, if the speculation proves true, 1) it could aid in identifying patients with a worse cognitive prognosis through circadian rhythm evaluation, enabling early intervention when necessary; 2) it may open up new possibilities for treating neurocognitive impairment in BD, for example, from a rhythmic perspective, such as brain synchronization; 3) it would provide stronger evidence supporting the pivotal role of circadian rhythm in BD pathophysiology [24].

Therefore, this study protocol was designed to bridge the current research gaps by investigating the predictability of neurocognitive functioning through circadian rhythm in BD. The objective of this study is to assess whether circadian rhythm could predict the long-term change in neurocognitive functioning among BD patients. It is hypothesized that circadian rhythm could predict the long-term change in neurocognitive functioning.

## Methods and analysis

### Setting and recruitment

**Figure 1.**
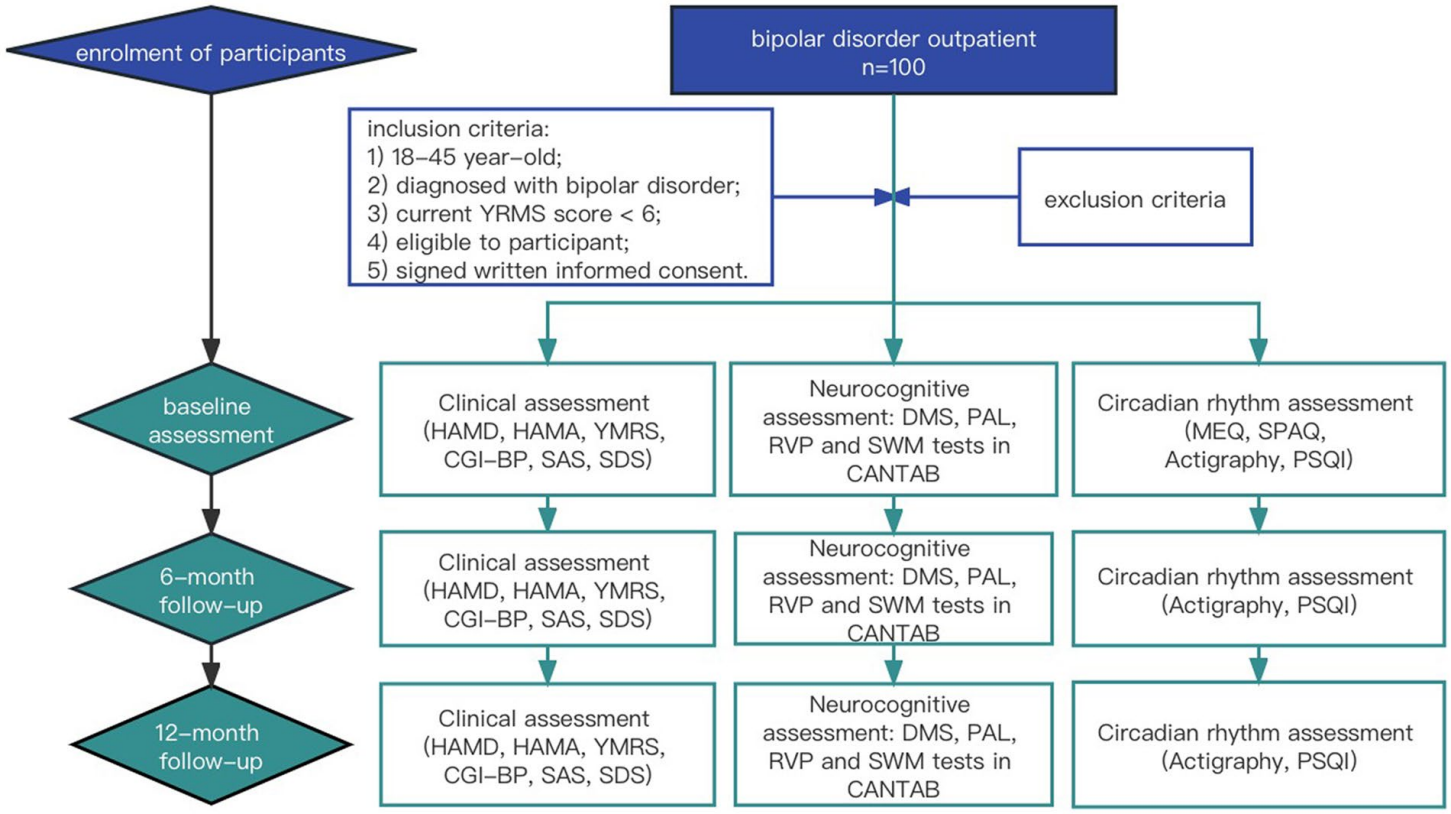
Flow diagram illustrating the procedures for participant enrollment and assessment.

This study follows a longitudinal cohort design, as depicted in Figure 1, with participant evaluations scheduled at baseline, 6-month follow-up, and 12-month follow-up after recruitment. Participants who miss the 6-month follow-up will still be eligible for the 12-month follow-up assessment. Ethical approval was obtained from the Research Ethics Committee of the First Affiliated Hospital of Chongqing Medical University (Approval Number: 20227001). Furthermore, this study has been registered with the Chinese Clinical Trial Registry under the registration code ChiCTR2200064922 on 21 October 2022. The study will be conducted in accordance with the Declaration of Helsinki [25].

Recruitment will be conducted at the Psychiatric Outpatient Department of The First Affiliated Hospital of Chongqing Medical University in China, which is one of the largest hospitals with an esteemed reputation in southwest China. Recruitment advertisements will be disseminated online, providing contact information for interested patients. Additionally, researchers will proactively contact potential participants based on electronic medical records and expert referrals. Participants will not receive any economic compensation, except for assistance with making outpatient appointments. The recruitment period will span from January 2023 to July 2023. Participants will have the autonomy to withdraw from the study, with reasons and dates recorded. No specific replacement plan is in place for participants who choose to drop out.

The inclusion criteria are as follows: 1) aged 18–45 years old, either female or male; 2) diagnosed with bipolar disorder according to the Diagnostic and Statistical Manual of Mental Disorders-5 (DSM-5), which will be confirmed by researchers using Structured clinical interview for DSM-5, research version (SCID-5-RV) [26,27]; 3) currently in depressive or remitted states, as indicated by a YRMS score < 6; 4) eligible to complete the required examinations; 5) signed written informed consent to participate in this study.

The exclusion criteria are as follows: 1) presence of other diseases that could severely impact circadian rhythm or neurocognitive functioning, such as amnesia, stroke, sleep apnea, obesity, hyperthyroidism, diabetes, brain injury, and cortisol or melatonin related endocrinopathy; 2) living conditions that might influence circadian rhythm or neurocognitive functioning, including engaging in shift work more than once a week, residing across time zones with a time difference exceeding two hours and a shifting frequency of more than once a month, and being in the perinatal or lactation period; 3) concurrent severe psychoactive substance use, defined as consuming alcohol more than twice a week, smoking over 20 cigarettes a day, or using psychedelics or other illegal substances; 4) currently experiencing strong suicidal ideation or attempt, or exhibiting high-risk impulsive, violent, or self-injurious behaviors necessitating immediate hospitalization or constant supervision; 5) having undergone electroconvulsive therapy within the last 6 months prior to recruitment.

The participants should not receive electroconvulsive therapy during the study; otherwise, their follow-up will be terminated. No other specific treatment requirements are in place.

### Assessment

Schedule of the enrolment and assessment was listed in Table 1.

**Table 1.** Schedule of enrolment and assessment.

Time point		Study periods		
		Baseline	6-month follow-up	12-month follow-up
Enrolment	Eligibility screen, informed consent, assessments	×		
Clinical assessment	HAMD-17, HAMA-14, YMRS, CGI-BP, SAS, SDS	×	×	×
Circadian rhythm assessment	MEQ, SPAQ	×		
	Actigraphy, PSQI	×	×	×
Neurocognitive assessment	DMS, PAL, RVP and SWM tests in CANTAB	×	×	×

Note: SAS: Zung’s self-rating anxiety scale; SDS: Zung’s self-rating depression scale; HAMD-17 : 17-item hamilton depression scale; HAMA-14:14-item hamilton anxiety scale; YMRS: young mania rating scale; CGI-BP: clinical global impressions-bipolar version; MEQ: morningness eveningness questionnaire; SPAQ: seasonal pattern assessment questionnaire; PSQI: Pittsburgh sleep quality index; DMS: delayed matching to sample; PAL: paired associates learning; RVP: rapid visual information processing; SWM: spatial working memory; CANTAB: Cambridge neuropsychological test automated battery.

#### Clinical assessment

Sociodemographic variables will be collected at baseline, including age, sex, height, weight, body mass index (BMI), ethnicity, education level, family history of mental illness, economic situation, handedness, history of tobacco and alcohol use and marriage status. We will also record the duration of illness, age of onset, subtypes of bipolar disorder, history of suicidal ideation and attempt, and history of psychotic symptoms.

For mood symptom monitoring at baseline, 6-month follow-up and 12-month follow-up, we will use the 17-item Hamilton Depression Scale (HAMD-17) and 14-item Hamilton Anxiety Scale (HAMA-14) which are interviewer-rating scales for depression and anxiety; Zung’s Self-Rating Depression Scale (SDS) and Zung’s Self-Rating Anxiety Scale (SAS) which are self-rating scales for depression and anxiety; and Young Mania Rating Scale (YMRS) which is an interviewer-rating scale for mania [28–33].

Other evaluation includes the Clinical Global Impressions-Bipolar Version (CGI-BP). CGI-BP is an interviewer-rating scale for disease severity and treatment efficacy [34].

#### Circadian rhythm assessment

The circadian rhythm variable consists of two components: subjective questionnaires and objective behavioral data. The questionnaires used include the Morningness Eveningness Questionnaire (MEQ), the Pittsburgh Sleep Quality Index (PSQI), and the Seasonal Pattern Assessment Questionnaire (SPAQ). The MEQ is a 19-item self-rating scale used to evaluate chronotype, with scores indicating evening, intermediate, or morning types [35]. The PSQI is an 18-item self-rating scale that assesses sleep quality and disturbances, and a validated Chinese version will be used [36,37]. The SPAQ is a widely used questionnaire for evaluating seasonal effects on mood and behavior. It includes a general seasonality score (GSS) that can be used to screen for seasonal affective disorder (SAD) [38,39]. Except for the SPAQ, all the aforementioned scales and questionnaires have already been validated or translated into Chinese versions.

On the day of evaluation, participants will be asked to wear a wrist-worn actigraph device (wGT3X-BT, v1.9.2, ActiGraph LLC) for 14 consecutive days and nights. The actigraph’s flashing light will be disabled throughout the monitoring period. The device will continuously record physical activity, sleep/wake information and sedentary time using a sampling frequency of 100 Hz. Participants will be instructed to continuously wear the actigraph except during activities such as showering, swimming, or any activity that would consistently expose the device to water for more than 30 min. Data retrieval and analyses will be performed using the ActiLife software program (version 6.13.4) with 60-second epochs [40]. The collected raw data will be stored and analyzed using publicly available algorithms. Non-wear time will be defined as 60 consecutive minutes of zeros with a 2-minute spike tolerance based on the default Wear Time Validation algorithm provided by Actilife 6.13.4. Any non-wearing epochs will be excluded from the analysis without any data imputation [41,42]. For a day to be deemed valid, it must have a minimum of 20 h of wearing time [42]. A participant will be considered valid if they have a minimum of 7 valid days, including at least 5 weekdays and 2 weekend days [35].

The circadian rhythms of sleep-wake activity will be calculated using Cole-Kripke Algorithm in Actilife [43,44]. We will retrieve sleep parameters such as Sleep Onset (SO), Total Sleep Time (TST), Wake after Sleep Onset (WASO) and Sleep Efficiency (SE). SO means the first minute that the algorithm scores ‘asleep’. TST equals to the total number of minutes scored as ‘asleep’. WASO means the total number of minutes the subject was awake after SO occurred. SE represents the number of sleep minutes divided by the total number of minutes the subject was in bed (i.e. the difference between the In-Bed and Out Bed time) [44].

The circadian rhythm parameters of physical activity will be calculated using the specialized GGIR package for R software [45]. Non-parametric circadian rhythm analysis will be employed. We aim to determine the intradaily variability (IV), interdaily stability (IS) and relative amplitude (RA) of the rest-activity rhythms [43,46,47]. IV refers to the consolidation of rest-activity states within a day, where higher values indicate greater fragmentation of the rhythm with more transitions between rest and active states [45]. IS describes the consistency of rest-activity patterns across different days, with higher values indicating greater stability [48]. RA represents the difference in activity between the most active 10-hour period (M10) and the least active 5-hour period (L5) and serves as a marker of amplitude strength [46]. Time of sedentary and moderate-to-vigorous physical activities (MVPA) will also be calculated based on validated activity cut-points in Actilife [35,49,50].

#### Neurocognitive assessment

The primary outcome will be a comprehensive cognitive composite score. Neurocognitive functioning will be evaluated using the Cambridge Neuropsychological Test Automated Battery (CANTAB), developed by Cambridge Cognition in Cambridge, UK (www.camcog.com) [51]. The tests have undergone validation in studies involving healthy human volunteers as well as various patient groups in the fields of behavioral and psychopharmacological research [52,53]. These tests will be administered on the same day as the actigraph wearing. The selected tests, including Delayed Matching to Sample (DMS), Paired Associates Learning (PAL), Rapid Visual Information Processing (RVP), and Spatial Working Memory (SWM), assess different cognitive domains [54–56]. DMS and PAL evaluate visual memory, RVP measures sustained attention, and SWM reflects executive function. By converting the results of these four tests into Z-scores, the scores of each participant on different cognitive domains can be derived, and a composite cognitive functioning score can be calculated [57,58].

### Sample size calculation

The sample size for this study was determined based on the primary hypothesis. To calculate the sample size, a review of relevant literature was conducted, and the PASS.15 computer software was used. Due to limited available reports, data from a previous study that evaluated changes in SWM between errors after 1 month of olanzapine treatment in 15 unipolar/bipolar depressive patients was used. In that study, the between errors at baseline and 1-month olanzapine treatment were 34 ± 24 and 21 ± 21, respectively (Mean ± Standard Deviation) [59].

With an assumed power of 0.80 and an alpha level of 0.05, it was determined that a cohort size of 41 at follow-up would achieve 80% power to detect a difference of −13 between the null hypothesis (both baseline and 12-month follow-up means of SWM errors are 34) and the alternative hypothesis (mean of 12-month follow-up is 21). The estimated standard deviations were 21 and 21 at baseline and 12-month follow-up, respectively, using a two-sample *t*-test [54]. Considering a potential dropout rate of 50% and the decision not to replace dropout participants, a total of 81 patients at baseline were deemed necessary. Finally, given the large number of outpatient receptions, we plan to recruit 100 participants.

Therefore, the study aimed to recruit a sample size of 100 patients at baseline to account for potential dropouts and achieve the desired statistical power.

### Statistical analysis

SPSS (version 26) will be used for the analysis of this study. Histograms will be used to determine the normality or approximate normality of the data. This will be achieved by combining them with the Shapiro-Wilk test [60]. Descriptive analyses will be performed to examine participant and disease characteristics, as well as data from clinical, circadian rhythm, and neurocognitive functioning assessments. Missing value analyses will be conducted with multiple imputation or expectation maximization methods to handle any missing data.

Spearman correlations will be employed to compare subjective and objectively assessed circadian rhythm patterns. Subjective assessments include MEQ, SPAQ, and PSQI results, while objective assessments will primarily rely on behavioral data.

Longitudinal analyses will involve calculating changes in SAS, SDS, HAMD, HAMA, and YMRS scores, and correlating these difference scores (baseline minus 6-month follow-up, baseline minus 12-month follow-up) with circadian rhythm parameters for each participant using Spearman correlation. Mixed regression models will be used to determine if changes in circadian rhythm significantly predict changes in neurocognitive functioning from baseline to 6-month follow-up and 12-month follow-up [61]. Stepwise adjustment of covariates will be employed to construct several regression models to explore the robustness of the relationship between circadian rhythm and neurocognitive functioning. Adjustments will be made for other factors that may influence these associations, such as disease duration, age, gender, current medication use, education level, and severity of emotional symptoms.

### Anticipated results

We anticipate that the circadian rhythm could predict the change of neurocognitive functioning among BD patients. More evidence of circadian rhythm importance for BD could be provided if the assumption holds.

## Discussion

The importance of circadian rhythm in BD has always been emphasized as BD is somehow rhythmic by itself in clinical oscillations among depressive, hypomanic/manic, and intermittent episodes [62]. The large genome-wide association study (GWAS) has found significant changes in gene expression around circadian rhythm pathways in BD population [63]. We hypothesized that disrupted circadian rhythm also plays a role in neurocognitive functioning impairment in BD. Currently, limited evidence could be cited to support such an opinion. Mice deficient in Vipr2, which participate in internal rhythm synchronization, exhibit cognitive deficits resembling schizophrenia [64,65]. Circadian clock-deficient cryptochrome knockout mice present with cognitive dysfunction and elevated anxiety [66]. Poor sleep quality is found related to poorer neuropsychological functioning in bipolar I disorder [67]. With RDoC highlighting circadian rhythm and cognitive evaluation in arousal/modulatory system and cognitive system separately, we can’t help speculating the role of circadian rhythm in neurocognitive impairment of BD, where we seek to better understand the correlation of circadian rhythm and neurocognitive functioning [11].

Still, we have not seen any study investigating the long-term neurocognitive predictability by circadian rhythm in BD patients. Thus, we designed such a study protocol where we included subjective rhythm questionnaires and objective actigraph data to represent the circadian rhythm. We plan to test our hypothesis from both cross-sectional and prospective perspectives. Specifically, to detect if the disrupted circadian rhythm is cross-sectionally correlated with worse neurocognitive functioning, if the subjectively measured circadian rhythm is comparable to objectively measured circadian rhythm, and if the circadian rhythm is predictive of long-term neurocognitive functioning changes.

This study is novel and intriguing due to the following advantages. Firstly, It is the first study protocol to establish the correlation between daily and seasonal circadian rhythms and neurocognitive functioning among BD patients. Secondly, it uses reliable yet less utilized techniques for evaluation accuracies, such as actigraph and CANTAB tests, with a combination of commonly recognized questionnaires for rhythm and mood evaluations. Thirdly, it is a prospective study lasting 12 months that fits in RDoC matrix, which could efficiently fill in the gap of long-term actigraph and cognitive functioning studies. Our study bears the potential to provide stronger evidence than a cross-sectional design and could enlighten similar designs guided by RDoC recommendations [8].

Our study also has some limitations. Initially, we do not explore the melatonin and cortisol levels fluctuations, which is the strongest indication of circadian rhythm [68]. Since we are not able to provide a laboratory condition for such repetitive samples, we are afraid that outpatients could not complete such a complicated measurement outside the hospital. Also, we did not use a sleep diary out of consideration that BD patients lack motivation with no economic compensation. Fortunately, there are some evidences supporting the comparability of cortisol and melatonin tests and sleep diary by actigraph data [69].

Secondly, this study is not a randomized controlled study, and a lot of facts might add bias to this study. This is a single-center study, and most patients will be recruited from our Bipolar Disorder Specialist Clinic, which is less presentative of real-world situations and might decrease the diversity of treatment procedures and medications. Moreover, to strengthen the validity and reliability of data, we minimized the amount of self-rating questionnaires, thus impeding more involvement of useful questionnaires such as the Athens Insomnia Scale, Biological Rhythms Interview of Assessment in Neuropsychiatry, 32-item Hypomania Checklist, Patient Health Questionnaire-9 and General Anxiety Disorder-7 [60,70–73]. Nevertheless, we argue that this is only an exploratory study. More strict study conditions could be set and adapted based on this small-sample study results.

Finally, there is little information for predicting our results due to lack of enough relevant studies. Therefore, the statistical methods might differ from our original design and should be adapted with our ultimate results.

In summary, this is a 12-month longitudinal cohort study protocol. We aimed to find evidence for circadian rhythm influence on neurocognitive functioning on BD patients. We hypothesize that circadian rhythm could predict the change of neurocognitive functioning. Our findings may provide additional information for BD pathophysiology, treatment targets and prediction. Larger and more homogeneous sample with more integrate design could be considered in future study.

## Data Availability

Not applicable.
